# Evaluation of an FDA approved library against laboratory models of human intestinal nematode infections

**DOI:** 10.1186/s13071-016-1616-0

**Published:** 2016-07-01

**Authors:** Jennifer Keiser, Gordana Panic, Roberto Adelfio, Noemi Cowan, Mireille Vargas, Ivan Scandale

**Affiliations:** Department of Medical Parasitology and Infection Biology, Swiss Tropical and Public Health Institute, Basel, Switzerland; University of Basel, Basel, Switzerland; Drugs for Neglected Disease initiative, Chemin Louis-Dunant 15, 1202 Genève, Switzerland

**Keywords:** FDA library, Soil-transmitted helminthiasis, Drug sensitivity assay, *Trichuris muris*, *Ancylostoma ceylanicum*, *Heligmosomoides polygyrus*

## Abstract

**Background:**

Treatment options for infections with soil-transmitted helminths (STH) - *Ascaris lumbricoides*, *Trichuris trichiura* and the two hookworm species, *Ancylostoma duodenale* and *Necator americanus* - are limited despite their considerable global health burden. The aim of the present study was to test the activity of an openly available FDA library against laboratory models of human intestinal nematode infections.

**Methods:**

All 1,600 drugs were first screened against *Ancylostoma ceylanicum* third-stage larvae (L3). Active compounds were scrutinized and toxic compounds, drugs indicated solely for topical use, and already well-studied anthelmintics were excluded. The remaining hit compounds were tested in parallel against *Trichuris muris* first-stage larvae (L1), *Heligmosomoides polygyrus* third-stage larvae (L3), and adult stages of the three species in vitro. In vivo studies were performed in the *H. polygyrus* and *T. muris* mice models.

**Results:**

Fifty-four of the 1,600 compounds tested revealed an activity of > 60 % against *A. ceylanicum* L3 (hit rate of 3.4 %), following incubation at 200 μM for 72 h. Twelve compounds progressed into further screens. Adult *A. ceylanicum* were the least affected (1/12 compounds active at 50 μM), while eight of the 12 test compounds revealed activity against *T. muris* L1 (100 μM) and adults (50 μM), and *H. polygyrus* L3 (200 μM). Trichlorfon was the only compound active against all stages of *A. ceylanicum*, *H. polygyrus* and *T. muris*. In addition, trichlorfon achieved high worm burden reductions of 80.1 and 98.9 %, following a single oral dose of 200 mg/kg in the *T. muris* and *H. polygyrus* mouse model, respectively.

**Conclusion:**

Drug screening on the larval stages of intestinal parasitic nematodes is feasible using small libraries and important given the empty drug discovery and development pipeline for STH infections. Differences and commonalities in drug activities across the different STH species and stages were confirmed. Hits identified might serve as a starting point for drug discovery for STH.

**Electronic supplementary material:**

The online version of this article (doi:10.1186/s13071-016-1616-0) contains supplementary material, which is available to authorized users.

## Background

Infections with soil-transmitted helminths (STH) - *Ascaris lumbricoides*, *Trichuris trichiura* and the two hookworm species, *Ancylostoma duodenale* and *Necator americanus -* are enormously prevalent and responsible for a main part of the global health burden associated with neglected tropical diseases. Approximately 1.45 billion people are infected globally with at least one STH species [1]. In 2013, 4.0 million disability adjusted life years (DALYs) were attributed to intestinal nematode infections [2]. Symptoms include malnutrition, iron deficiency, anemia, intestinal obstruction, chronic dysentery, rectal prolapse, and poor weight gain [3]. To date, the most cost-effective public health strategy is to control morbidity by periodically administering anthelmintic drugs of mainly albendazole and mebendazole, on a large-scale. In 2014, 138.2 and 258 million preschool-aged and school-aged children, respectively, were treated with the benzimidazoles in endemic countries [4]. This number will increase considerably over the next years, since more than 800 million children require annual treatment with anthelmintic drugs [4]. Drug resistance is a serious concern in light of the enormous drug pressure. In addition, the spectrum of activity of the benzimidazoles is sub-optimal; albendazole and mebendazole have only low efficacy against the intestinal nematode species *T. trichiura* when administered at single doses [5]. It is therefore imperative that the drug discovery pipeline produces viable alternatives.

Drug discovery for most of the STH is truly neglected. For example, in 2014 as little as 678,299 and 171,197 USD were invested in drug discovery research for hookworm and *T. trichiura* infections, respectively (https://gfinder.policycures.org/PublicSearchTool). To our knowledge there are no novel drug candidates for soil-transmitted helminthiasis in the drug development pipeline.

In order to contribute to research and development efforts for these neglected tropical diseases, a series of libraries were screened for activity against hookworm and/or whipworm in the framework of a Gates-funded project in collaboration with the Drugs for Neglected Disease *initiative*. Here we present our results with an openly available FDA library. All 1,600 drugs were first screened against the larval stages of *A. ceylanicum.* Active compounds were examined and toxic compounds, drugs indicated solely for topical use and already well-studied anthelmintics were excluded. The remaining hit compounds were tested in parallel against adult *A. ceylanicum* and against *T. muris* L1 and *Heligmosomoides polygyrus* L3 and adults in vitro. In vivo studies were performed in the *H. polygyrus* and *T. muris* mice models.

## Methods

### Animals

Three-week-old male Syrian Golden hamsters were purchased from Charles River (Sulzfeld, Germany). Four-week-old female NMRI mice and female C57BL/10ScSnOlaHsd mice (age 3 weeks) were purchased from Harlan Laboratories (Horst, The Netherlands and Blackthorn, United Kingdom, respectively). All animals were kept in macrolon cages under environmentally-controlled conditions (temperature: 25 °C, humidity: 70 %, light/dark cycle 12 h/12 h) and had free access to water (municipal tap water supply) and rodent food. Rodents were allowed to acclimatize for one week before infection. The current study was approved by the local veterinary agency, based on Swiss cantonal and national regulations (permission no. 2070).

### Drugs

The FDA Pharmakon compound library was purchased from MicroSource Discovery Systems, Inc. (USA). Compounds (10 mM, dissolved in DMSO) were stored at -80 °C until use. Levamisole and ivermectin, used for the in vitro controls, were purchased from Fluka and Sigma-Aldrich, respectively (Buchs, Switzerland), dissolved at 10 mM stock solutions in DMSO, and stored at -20 °C until further use. For the in vivo studies, bitoscanate, chlorcyclizine HCl, clemastine, dicyclomine HCl, drofenine HCl, ethopropazine HCl, lansoprazole, metformin HCl, morantel, natamycin, trichlorfon, and trimipramine maleate were purchased from Sigma-Aldrich.

### In vitro tests on *A. ceylanicum* L3

*A. ceylanicum* larvae (L3) were obtained by filtering the feces of infected hamsters and cultivating the eggs for 9 days in the dark at 24 °C. L3 were washed in penicillin and streptomycin-supplemented tap water and kept under refrigeration until used. For the drug assay, 40 L3 were placed in each well of a 96-well plate for each compound, in duplicate. Worms were incubated with 200 μM of the test drug and culture medium, which was composed of 100 μl HBSS medium (Hanks’ Balanced Salt Solution Modified; GIBCO, Lucerne, Switzerland) supplemented with 25 μg/ml amphotericin B, 100 U/ml penicillin, and 100 μg/ml streptomycin (Sigma-Aldrich). Worms incubated with culture medium and 1 % DMSO served as negative controls. Wells containing larvae, medium, and 200 μM of levamisole served as positive controls. The plates were kept at room temperature and in the dark for up to 72 h, after which the drug effect was evaluated. To do so, first the total number of L3 per well was determined. Then, 100 μl of hot water (≈80 °C) was added to each well and the larvae that responded to this stimulus (the moving L3) were counted. The proportion of larval death was determined. A cut-off of 60 % was used to define activity, hence compounds achieving > 60 % larval death progressed to further testing.

### In vitro tests on *A. ceylanicum* adult worms

Hamsters were infected *per os* with 150 *A. ceylanicum* L3. Three weeks post-infection, hamsters were euthanized with CO_2_ and the worms were collected from the intestine. For each compound, three *A. ceylanicum* adults were placed in each well of a 24-well plate, using 2 wells per compound. Worms were incubated in the presence of 50 μM of the drug to be tested, and culture medium, which was composed of HBSS medium supplemented with 10 % Fetal Calf Serum (FCS) (Connectorate AG, Dietikon, Switzerland), 25 μg/ml amphotericin B, 100 U/ml penicillin, and 100 μg/ml streptomycin. Worms incubated in 1 % DMSO in culture medium served as control. As a positive control, ivermectin was used at a concentration of 50 μM. Worms were kept in an incubator at 37 °C and 5 % CO_2_ for up to 72 h. Thereafter, the condition of the worms was microscopically evaluated using a viability scale from 3 (normal activity and no tegument alteration) to 0 (dead, completely granulated).

### In vitro tests on *T. muris* L1 worms

Female C57BL/10ScSnOlaHsd mice were infected *per os* with 200–250 *T. muris* eggs showing around 90–95 % embryonation (checked microscopically). After 7 weeks, *T. muris* eggs were collected from the feces of the infected mice by the flotation method using saturated NaCl solution in Milli-Q water. *T. muris* eggs were stored in Milli-Q water in the dark for 3 months at 23–25 °C, until the eggs were embryonated. The hatching process to obtain *T. muris* L1 has been described elsewhere [6]. For the assay, 40 L1 larvae were placed in each well of a 96-well plate. Worms were incubated in the presence of 100 μl RPMI 1640 medium with 12.5 μg/ml amphotericin B, 500 U/ml penicillin, 500 μg/ml streptomycin, and 100 μM of the drug to be tested. Each drug was tested in duplicate. L1 larvae incubated with culture medium and 1 % DMSO served as a negative control and larvae incubated with levamisole at a concentration of 100 μM were included as a positive control. The assay was kept in an incubator at 37 °C and 5 % CO_2_ for 24 h. At 24 h, the total number of L1 larvae per well was counted. The larvae were then stimulated with 100 μl hot water (≈80 °C) and the moving L1 larvae were counted.

### In vitro assay with *T. muris* adult worms

Seven weeks post-infection of female C57BL/10ScSnOlaHsd mice, *T. muris* adult worms were collected from the intestines. Three *T. muris* adult worms were placed in each well of a 24-well plate. Worms were incubated with culture medium and 50 μM of the drug to be tested. Each compound was tested in duplicate. *Trichuris muris* adult worms incubated with 1 % DMSO and culture medium served as control. As positive control, levamisole was used at a concentration of 50 μM. Worms were kept in an incubator at 37 °C and 5 % CO_2_ for 72 h and were subsequently microscopically evaluated using a viability scale from 3 (normal activity and no tegument alteration) to 0 (dead, completely granulated).

### *Heligmosomoides polygyrus* L3 in vitro studies

Female NMRI mice were infected with 80 *H. polygyrus* L3 *per os. H. polygyrus* eggs were obtained from infected feces. The eggs were then placed on agar and, after 9 days in the dark at 24 °C, the L3 larvae hatched. For the drug assay, 40 L3 larvae were placed in each well of a 96-well plate. Worms were incubated in the presence of 100 μl RPMI 1640 medium, supplemented with 0.63 μg/ml amphotericin B, 500 U/ml penicillin, 500 μg/ml streptomycin, and 100 μM of the drug to be tested. Each drug was tested in duplicate. Worms incubated with 1 % DMSO and culture medium served as a negative control and wells containing larvae, medium and 100 μM levamisole served as a positive control. The plates were kept at room temperature for up to 72 h. To assess the effect of the compound, the total number of L3 larvae per well was counted, the larvae were stimulated with 100 μl hot water (≈80 °C), and the moving L3 were counted.

### In vitro assay with *H. polygyrus* adults

Female NMRI mice were infected with 80 *H. polygyrus* L3 *per os*. Two weeks post-infection, mice were dissected and three adult worms were placed in each well of a 24-well plate. Worms were incubated with culture medium and 50 μM of the test drug. Each compound was tested in duplicate. Adult worms incubated with medium only and 50 μM levamisole and ivermectin served as negative and positive control, respectively. Worms were kept in an incubator at 37 °C and 5 % CO_2_ for 72 h and, subsequently, were microscopically evaluated using a viability scale from 3 to 0.

### *Trichuris muris* in vivo studies

C57BL/10ScSnOlaHsd mice were infected with 200 embryonated *T. muris* eggs *per os*. The drinking water for the mice contained dexamethasone (1 mg/ml) in order to suppress their immune system to avoid rejection of the infection. At 42 days post-infection, the stool was collected and filtered to check for the presence of eggs. Infected mice were placed in individual cages. The compounds were administered by gavage at dosages of 10–400 mg/kg (guided by the LD_50_ values of the drugs). The dose volume was adjusted according to the mouse’s body weight, which was assessed for each mouse directly prior to treatment. Four to six untreated mice served as controls. After 72 h, the stool was collected from each cage to check and count the dead worms released through the stool. Six days post-treatment, animals were killed by the CO_2_ method and the gastrointestinal tract was collected. The intestine was dissected and adult worms were collected and counted.

### *Heligmosomoides polygyrus* in vivo studies

NMRI mice were infected with 80 *H. polygyrus* L3 *per os*. Fourteen days post-infection, mice were treated orally with the test drugs at dosages of 10–400 mg/kg. Four to six untreated mice served as controls. Ten days post-treatment, animals were killed by the CO_2_ method, and the gastrointestinal tract was collected. The intestine was dissected and adult worms were collected and counted.

### Statistics

For the in vitro drug sensitivity assays, all viability scores/larval survival counts were averaged across replicates and normalized to the average viability scores/larval survival counts of the control wells (Microsoft Office Excel 2010) using the following formula: Drug effect (%) = 100 % – [100 % * (No. of live larvae alive/Total No. of larvae) drug/(No. of live larvae/Total No. of larvae) control]. To calculate the drug effect in in vivo studies, the worm burden (WB) of treated animals was calculated and compared with the worm burden of control mice, which were infected simultaneously but were not treated. The worm burden reduction (WBR) was calculated as follows: WBR (%) = 100 % – (100 % * WB treatment/WB control). The worm expulsion rates were calculated as follows: (c/d) × 100, where c is the total number of expelled worms in a treated group and d is the total worm count (expelled worms as well as worms present in the gut counted following dissection) of the same group. The Kruskal-Wallis test (Statsdirect, statistical software version 2.8.0) was used to determine statistical significance of WBRs at a level of 0.05.

## Results

### In vitro studies

**Fig. 1:**
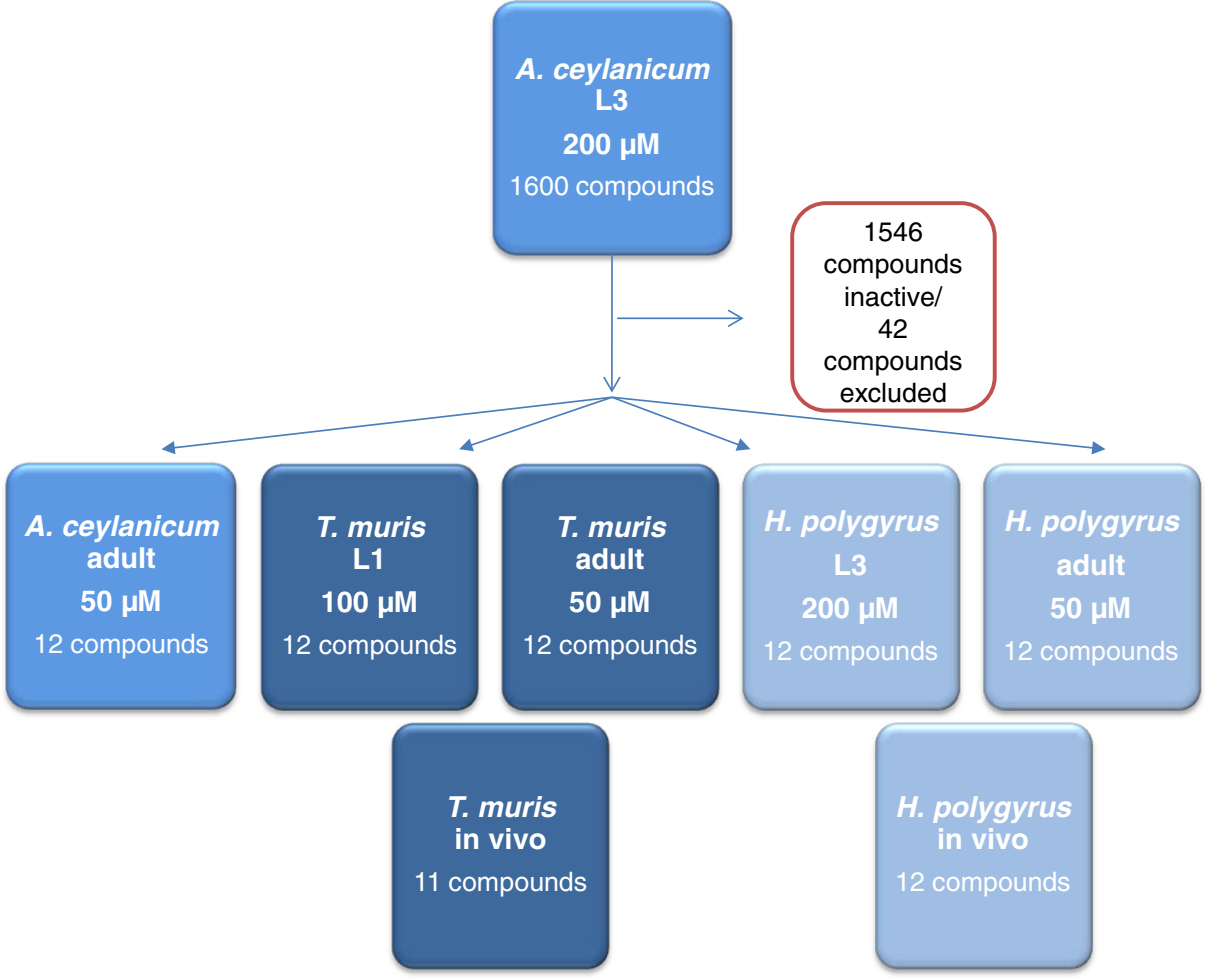
Screening flow applied testing a library of 1600 FDA approved compounds against *A. ceylanicum*, *T. muris* and *H. polygyrus*

The study flow is presented in Fig. 1. From 1,600 compounds tested, 54 compounds revealed an activity of > 60 % against *A. ceylanicum* L3 (hit rate of 3.4 %), following incubation at 200 μM for 72 h. Twenty of these compounds killed all L3. Results are summarized in Table 1. The activities of the standard drugs, levamisole and ivermectin, (also included in the library) are presented as reference. Both drugs resulted in death of all *A. ceylanicum* L3.

**Table 1:** In vitro activity profile of 56 FDA compounds (including 2 reference compounds) against *A. ceylanicum* and *T. muris* adult and larval stages

Compound	Drug effect (%)					
	*A. ceylanicum* L3 200 μM (72 h)	*H. polygyrus* L3 200 μM (72 h)	*T. muris* L1 100 μM (24 h)	*A. ceylanicum* adult 50 μM (72 h)	*H. polyygrus* adult 50 μM (72 h)	*T. muris* adult 50 μM (72 h)
Levamisole	100	100	100	0	56	100
Ivermectin	100	93	0	100	41	0
Abamectin	100	ND, anthelmintic				
Amitriptyline HCl	91	ND, toxic				
Apomorphine HCl	72	ND, toxic				
Bifonazole	67	ND, topical application				
Bitoscanate	100	20	100	28	34	100
Carbachol	89	ND, topical application				
Chlorambucil	95	ND, toxic				
Chlorcyclizine HCl	78	77	78	23	30	31
Chlorhexidine dihydrochloride	61	ND, topical application				
Chloroxylenol	100	ND, topical application				
Chlorpromazine	100	ND, toxic				
Chlorprothixene HCl	100	ND, toxic				
Chlorpyrifos	97	ND, toxic				
Cisplatin	87	ND, toxic				
Clemastine fumarate	76	91	2	47	30	100
Clomiphene citrate	80	ND, toxic				
Coumophos	100	ND, toxic				
Cyproheptadine HCl	88	ND, toxic				
Dexlansoprazole	88	35	1	47	17	55
Diatrizoic acid	81	ND, toxic				
Dibenzothiophene	100	ND, topical application				
Dicyclomine HCl	71	88	7	42	100	93
Dimpylate	100	ND, toxic				
Doramectin	100	ND, anthelmintic				
Doxepin HCl	64	ND, toxic				
Drofenine HCl	62	87	100	44	20	100
Dyclonine HCl	100	ND, topical				
Eprinomectin	100	ND, anthelmintic				
Ethopropazine HCl	100	89	100	33	100	100
Fenbendazole	66	ND, anthelmintic				
Fenthion	97	ND, toxic				
Ftaxilide	73	ND, commercially not available				
Hexachlorophene	100	ND, topical				
Histamine dihydrochloride	62	ND, topical				
Mebendazole	78	ND, anthelmintic				
Metformin HCl	100	11	100	50	27	75
Metitepine mesylate	70	ND, toxic				
Morantel citrate	60	100	100	47	80	94
Moxidectin	100	ND, anthelmintic				
Natamycin	95	14	100	53	10	61
Phenothiazine	98	ND, toxic				
Phenylmercuric acetate	100	ND, topical				
Pramoxine HCl	100	ND, topical				
Pyrantel pamoate	91	ND, anthelmintic				
Selamectin	79	ND, anthelmintic, topical				
Tetramizole HCl	100	ND, anthelmintic				
Thiabendazole	93	ND, anthelmintic				
Thonzonium bromide	98	ND, topical				
Trichlorfon	100	89	100	100	79	100
Triclosan	98	ND, topical				
Triflupromazine HCl	83	ND, toxic				
Trimeprazine tartrate	64	ND, toxic				
Trimipramine maleate	62	79	99	7	23	75
Zoxazolamine	98	ND, toxic				

*Abbreviation*: *ND* Not done

Of the 54 compounds, 12 compounds have a topical indication and 19 drugs are toxic or are associated with severe adverse events. Twelve drugs (including levamisole and ivermectin) are well-known anthelmintics and, with exception of bitoscanate and morantel (for which little information on the efficacy against these parasites is available), were therefore not considered for further testing in the framework of this work. One drug, ftaxilide was not commercially available. Hence, 12 compounds progressed into further screens. Against adult *A. ceylanicum,* only trichlorfon resulted in death of the worms, while the remaining 11 compounds revealed low activity (< 60 % activity). Eight of the 12 compounds affected *H. polygyrus* L3, with an activity of > 75 %. Adult *H. polygyrus* showed slightly higher sensitivity to the drugs as compared to adult *A. ceylanicum*, with dicyclomine HCl and ethopropazine HCl resulting in death of all worms, and trichlorfon and morantel revealing an activity of 79 and 80 %, respectively.

Against *T. muris* L1, nine of the 12 test compounds showed an activity ranging from 78–100 %, at a concentration of 100 μM after 24 h of incubation. Six of these compounds caused death of all worms. The parallel tests on adult *T. muris* yielded nine compounds with high activity (75–100 %) against *T. muris* adults, of which five compounds were lethal. There was a good correlation between larval and adult activity: two compounds were active against adult *T. muris*, but not active against L1. Conversely, one compound showed no activity against adult *T. muris* despite being active against L1 (Table 1).

Trichlorfon was the only compound active against all stages of *A. ceylanicum*, *H. polygyrus* and *T. muris*.

### In vivo studies against *H. polygyrus*

Identified hits from in vitro studies (*n* = 12) were further evaluated for in vivo proof of principle. To maximize chances of success, compounds were evaluated at high doses. Single administrations were favored to mimic dosing regimens used in mass drug administration programs for treatment of STH infections with the current standard of care.

Low activity was observed for chlorcyclizine HCl, clemastine, dicyclomine HCl, drofenine HCl, ethopropazine HCl, lansoprazole, metformin HCl and trimipramine maleate, with WBRs below 41 %. In mice treated with a single oral dose of 400 mg/kg natamycin, a WBR of 59.7 % was observed. The highest activities were observed with bitoscanate and trichlorfon. Both dosages of bitoscanate (100 and 50 mg/kg) resulted in significant WBRs of 100 and 98.8 %, respectively. A WBR (98.9 %) was achieved with a single oral dose of 200 mg/kg trichlorfon, curing three of four treated mice (Table 2). At 100 mg/kg trichlorfon, the calculated WBR was still significant (81.9 %).

**Table 2:** In vivo studies against *H. polygyrus* and *T. muris*

Treatment	Dose (mg/kg)	*T. muris*				*H. polygyrus*		
		No. of mice cured/investigated	Average worm number	Worm burden reduction (%)	Worm expulsion rate (%)	No. of mice cured/investigated	Average worm number	Worm burden reduction (%)
Control a	–	0/6	170.95	–	–	–	---	---
Control b	–	0/4	163.5	–	–	–	---	---
Control c	–	0/4	197.3	–	–	–	---	---
Control 1						0/8	61.3	---
Control 2						0/8	21.1	
Control 3						0/12	47.0	
Bitoscanate^a,3^	100	0/3*	70.5	58.8	0.3	4/4	0	100
Bitoscanate^2^	50	ND	–	–	–	3/4	0.25	98.8^¶^
Chlorcyclizine HCl^c,1^	100	0/3	115.0	41.7		0/4	39.5	35.6
Clemastine^b,1^	100	0/3	130.7	20.1	2.5	0/4	84.8	0
Dicyclomine HCl^b,1^	400	0/3	245.5	0	39.1	0/4	36.8	40.1
Drofenine HCl^a,1^	400	0/3	267.6	0	3.1	0/4	54.0	12.0
Ethoproprazine HCl^b,1^	200	0/3	368.0	0	6.7	0/4	45.5	25.8
Lansoprazole^1^	400	ND	–	–	–	0/4	43.0	29.9
Metformin HCl^a,1^	400	0/3	140.6	17.7	0.6	0/4	80.0	0
Morantel^c,1^	400	0/3**	367.0	0	0	0/2**		
Natamycin^a,1^	400	0/3***	–	–	–	2/4	24.8	59.7
Trichlorfon^c,1^	200	1/3	34.0	80.1^¶^	67.6	3/4	0.5	98.9^¶^
Trichlorfon^a^	100	0/3	121.0	28.8	3.3	2/4	8.5	81.9^¶^
Trimipramine maleate^c,1^	10	0/3	113.3	42.6	0.1	0/4	71.5	0

Each * indicates a mouse that died. Superscripts 1–3 and a-c refer to the respective control groups ^¶^ *P* < 0.05

### In vivo studies against *T. muris*

Nine of the 11 compounds tested in vivo revealed a low activity against *T. muris* (WER < 7 % and WBRs < 45 %)*.* Natamycin and morantel were not tolerated by the mice and five out of six mice died within 24 h post-treatment. Treatment of infected mice with bitoscanate achieved a WBR of 42 %, however, one of the mice died two days after treatment. The highest activity was observed with trichlorfon administered at 200 mg/kg, which resulted in a WBR of 80 % and a WER of 67 %. Halving the dose to 100 mg/kg resulted in a low WBR and WER (28.8 and 3.3 %, respectively) (Table 2).

## Discussion

Drug repurposing is the key drug discovery strategy for human helminth infections, since anthelmintic drug discovery and development has languished [7]. Veterinary anthelmintics have obviously been the most attractive candidates for cross-over development for the treatment of human STH infections, since the regulatory standards for veterinary drugs are compatible with those for human drugs and a track-record of their use in animals exists [8]. Indeed, all of the few anthelmintics currently used for humans stem from veterinary medicine. In the present work, we applied a broader repurposing strategy, by evaluating 1,600 compounds from the FDA library, which contains approved drugs over a range of indications.

While a handful of studies employing target screening approaches have been conducted to identify new pharmacophores for the treatment of human STH infections [9], phenotypic screening of selected libraries on parasitic nematodes is basically non-existent. However, whole-organism screens have several advantages over target-based screening, since they are not constrained to single targets (which are not well characterized for helminths) [10]. Indeed, phenotypic approaches are more successful for small-molecule, first-in-class medicines than target based approaches [11]. High throughput assays with *Caenorhabditis elegans* often serve as a substitute for helminth phenotypic assays, because most assays for parasitic nematodes are laborious and low throughput. However, the correlation of activity against *C. elegans* to parasitic nematodes is not universal, with for example albendazole lacking activity against *C. elegans* [12].

We observed a hit rate of 3.4 % against *A. ceylanicum* larvae, our primary screen, with hits ranging across a large spectrum of indications. For comparison, a hit rate of ~7.6 % was observed against *S. mansoni* schistosomula using the same library and also encompassing a wide range of compound indications [13]. As highlighted already for the *S. mansoni* screen [13], it was disappointing that many of the hits were unsuitable for further testing, notably due to documented toxicity or restriction to topical use. In addition, we excluded the common anthelmintics (e.g. mebendazole, pyrantel). Selected compounds were next tested against a panel of helminths, namely *A. ceylanicum* adults, *H. polygyrus* larvae and adults, as well as *T. muris* larvae and adults. A few observations are offered for discussion. First, *A. ceylanicum* L3 were selected for the primary screen since, compared to adult stages, their use offers many advantages, mainly in terms of ethical considerations, numbers available, and ease of provision [14]. However, larval stages may not always be equally as sensitive as the target parasite stage, the adult worms. For example, the veterinary anthelmintic, monepantel, lacks activity against *A. ceylanicum* L3, while it is active against the adult worm [15]. Hence, larval-based assays should be validated in terms of how larval sensitivity compares to the sensitivity of adult worms. Additional file 1: Table S1 summarizes the activity of all 54 *A. ceylanicum* L3 active drugs (including topical and toxic drugs) against adult *A. ceylanicum*, *T. muris* L1 and adult *T. muris*. In general, *A. ceylanicum* L3 were more sensitive in our assay to the test compounds than the adult stages. Increased sensitivities of larval forms over adult worms have been reported earlier including for *S. mansoni* schistosomula [16]. Though a high false positive rate (larval activity does not always translate to adult activity) is not optimally cost-effective, the risk of losing interesting compounds is minimal, and in any case, larval pre-screens are still more cost-effective and more ethical than conducting the entire screen on adult stage worms. However, for *T. muris* this trend could not be confirmed (Additional file 1: Table S1). Adult *T. muris* were equally or even more sensitive than *T. muris* L1. Second, to our knowledge, we have for the first time compared the sensitivities of a small series of test drugs on larval and adult stages of the two hookworm species. The *H. polygyrus* model is widely used to study intestinal nematode infections, as it is easy to maintain in the laboratory and far more cost-effective than other laboratory hookworm rodent models [17]. The activity of compounds on *A. ceylanicum* L3 and *H. polygyrus* L3 was similar (only bitoscanate, dexlansoprazole, metformin and natamycin lacked activity against *H. polygyrus* L3). Similarly, the *A. ceylanicum* adult screen mirrored the adult *H. polygyrus* screen (except for dicyclomine HCl and ethopropazine, which showed activity against adult *H. polygyrus*). Hence, our results with a small panel of compounds support the use of *H. polygyrus* in the framework of anthelmintic drug discovery.

Only two compounds revealed activity in vivo, namely trichlorfon and bitoscanate. In addition, a moderate activity was observed with natamycin against *H. polygyrus* (two out of four mice cured), while the drug was not tolerated by *T. muris* infected mice. The low in vivo activity of many of the in vivo tested drugs might not be surprising, as bioavailable drugs from the FDA library of 1,600 approved drugs may not have the ideal profile for in vivo activity. Though there are exceptions (e.g. levamisole, ivermectin or albendazole) an ideal treatment for STH, particularly *Trichuris* spp. infections should be only poorly absorbed in order to target worms sitting in the gastrointestinal tract.

In vivo, trichlorfon revealed high activity against *H. polygyrus* and *T. muris*, while bitoscanate showed high activity against hookworm but was moderately active against *T. muris*. The organophosphate trichlorfon is mainly used as an ectoparasiticide [18]. However, it is also known for its antischistosomal properties [trichlorfon (metrifonate) was marketed for the treatment of *S. haematobium* prior to the advent of praziquantel] [19] and is applied for the control of intestinal nematode parasites of cattle and sheep. Trichlorfon is particularly used for nematodes that have developed resistance to other commonly-used anthelmintics [20]. In the past years, it was investigated for the treatment of Alzheimer’s disease. Metrifonate, at various fixed and loading doses, was associated with significant cognitive improvement compared to placebo, where the slow-release break-down byproduct, 2,2-dichlorovinyl dimethyl phosphate (DDVP), is supposed to be the active component [21, 22]. The broad activity of metrifonate is perhaps not surprising considering its mechanism of action; metrifonate is a cholinergic drug, acting as an irreversible non-selective acetylcholinesterase and butyrylcholinesterase inhibitor [21]. There is a history of active cholinergic anthelmintics including pyrantel and levamisole, which are both selective nAChR agonists, and ivermectin and moxidectin, which are modulators of glutamate-gated ion channels and nAChRs [23]. Emerging cholinergic anthelmintics, monepantel, tribendimidine and derquantel [24–26] have different nAChR subtype selectivities.

Bitoscanate was widely used prior to the advent of the benzimidazoles for the treatment of hookworm infections [27, 28]. Despite its history of use, the literature is very scarce. Bitoscanate belongs to the broad isothiocyanate class of anthelmintic compounds, which occur widely in nature, particularly in plants of the mustard (Brassicacae*)* family [29]. In India, clinical trials with thousands of hookworm-infected patients were conducted, which revealed a high efficacy of the drug. Conflicting results were observed with regard to *Trichuris* spp.; while a good trichuricidal activity was observed in some settings [30], the high efficacy was not consistently seen [28]. Mild and transient adverse events were reported in the large number of studies done with bitoscanate [28]. However, in the US bitoscanate is listed as an extremely hazardous substance (http://nj.gov/health/eoh/rtkweb/documents/fs/2172.pdf) which, when released in certain amounts in the environment, may be of immediate concern to the community. A related compound, amoscanate, was found to be effective against hookworm and schistosomes in humans, but was accompanied with severe liver toxicity in laboratory animals at high doses [31, 32]. However, another related compound, nitroscanate, is widely employed in veterinary medicine to treat roundworm, hookworm and tapeworms [33]. Currently, the isothiocyanates are researched for activity against intestinal bacteria and cancer [34, 35].

The same library was screened against *S. mansoni* larval stage and adult worms [13] with a very different set of hits. When compared, 15 (27.8 %) of the compounds identified as hits in this screen were also hits against *S. mansoni* newly-transformed schistosomula (NTS) (abamectin, chlorprothixene hydrochloride, clomiphene citrate, doramectin, eprinomectin, hexachlorophene, metformin hydrochloride, metitepine mesylate, moxidectin, natamycin, phenylmercuric acetate, selamectin, thonzonium bromide, trichlorfon and triflupromazine hydrochloride). The vast majority of these common hits were either toxic or for topical use only. However, it is also noted that the macrocyclic lactones, a group that acts on glutamate-gated ion channels [36], in general appear to be active against both *S. mansoni* and hookworm.

## Conclusions

In conclusion, we tested the FDA library of 1,600 approved drugs using a screening cascade with the larval stages of the parasitic nematode, *A. ceylanicum,* as a primary model. Our work has demonstrated that drug screening on the larval stages of intestinal parasitic nematodes is feasible using small libraries and shows differences and commonalities in drug activities across the different STH species and stages. A moderate hit rate of 3.4 % was observed with the FDA library of approved drugs, however many of the active drugs consisted of toxic compounds, compounds for topical use, or anthelmintics. Trichlorfon and bitoscanate were the only active compounds in in vivo studies; however both drugs have clear limitations. Nonetheless, structurally related pharmacophores might serve as a starting point for drug discovery for STH infections.

## Additional file

Additional file 1: Table S1. In vitro activity profile of 56 FDA compounds against *A. ceylanicum* and *T. muris* larval and adult stages (including topical and toxic drugs). (DOCX 52 kb)
