# Supplementary material for: Evaluation of an FDA approved library against laboratory models of human intestinal nematode infections
Source: Parasit Vectors. 2016 Jul 1;9:376. doi: 10.1186/s13071-016-1616-0 (PMC4929775; doi:10.1186/s13071-016-1616-0)
Supplement: Additional file 1: Table S1. — In vitro activity profile of 56 FDA compounds against A. ceylanicum and T. muris larval and adult stages (including topical and toxic drugs). (DOCX 52 kb) [file 13071_2016_1616_MOESM1_ESM.docx]

**Supplementary Table 1** In vitro activity profile of 56 FDA compounds against *A. ceylanicum* and *T. muris* larval and adult stages (including topical and toxic drugs)

| Compound | **Drug effect (%)**  ***A. ceylanicum* L3**  200 µM (72 h) | **Drug effect (%)**  ***A. ceylanicum* adult**  50 µM (72 h) | **Drug effect (%)**  ***T. muris* L1**  100 µM (24 h) | **Drug effect (%)**  ***T. muris* adult**  50 µM (72 h) |
| --- | --- | --- | --- | --- |
| Levamisole | 100 | 0 | 100 | 100 |
| Ivermectin | 100 | 100 | 0 | 0 |
| Abamectin | 100 | 100 | 20.0 | 100 |
| Amitriptyline HCl | 91.3 | 13.3 | 100 | 77.8 |
| Apomorphine HCl | 71.9 | 47.2 | 5.1 | 100 |
| Bifonazole | 67.0 | 94.4 | 100 | 100 |
| Bitoscanate | 100 | 27.8 | 100 | 100 |
| Carbachol | 88.6 | 80.0 | 100 | 97.2 |
| Chlorambucil | 95.1 | 52.8 | 1.5 | 53.3 |
| Chlorcyclizine HCl | 77.5 | 23.3 | 78.2 | 30.6 |
| Chlorhexidine dihydrochloride | 61.3 | 20.0 | 98.2 | 100 |
| Chloroxylenol | 100 | 25.0 | 2.3 | 60.0 |
| Chlorpromazine | 100 | 40.0 | 100 | 100 |
| Chlorprothixene HCl | 100 | 36.7 | 100 | 100 |
| Chlorpyrifos | 96.8 | 100 | 0 | 0 |
| Cisplatin | 87.2 | 63.9 | 6.5 | 33.3 |
| Clemastine fumarate | 76.2 | 47.2 | 1.5 | 100 |
| Clomiphene citrate | 80.4 | 52.8 | 4.7 | 100 |
| Coumophos | 100 | 66.7 | 2.5 | 11.1 |
| Cyproheptadine HCl | 87.5 | 38.9 | 100 | 100 |
| Dexlansoprazole | 88.0 | 47.2 | 1.3 | 54.8 |
| Diatrizoic acid | 80.8 | 16.7 | 58.3 | 57.1 |
| Dibenzothiophene | 100 | 66.7 | 21.2 | 19.4 |
| Dicyclomine HCl | 71.4 | 41.7 | 6.8 | 93.0 |
| Dimpylate | 100 | 72.2 | 0 | 22.2 |
| Doramectin | 100 | ND | 0 | 100 |
| Doxepin HCl | 63.7 | 13.9 | 6.8 | 47.6 |
| Drofenine HCl | 62.3 | 44.4 | 100 | 100 |
| Dyclonine HCl | 100 | 47.2 | 2.3 | 70.8 |
| Eprinomectin | 100 | 88.9 | 3.6 | ND |
| Ethopropazine HCl | 100 | 33.3 | 100 | 100 |
| Fenbendazole | 65.8 | 52.8 | 0 | 16.7 |
| Fenthion | 96.7 | 72.2 | 0 | 63.0 |
| Ftaxilide | 72.8 | 25.0 | 0 | 16.7 |
| Hexachlorophene | 100 | ND | 100 | ND |
| Histamine dihydrochloride | 62.2 | 33.3 | 2.3 | 47.2 |
| Ivermectin | 96.1 | 100 | 0 | 25.0 |
| Levamisole HCl | 100 | 29.2 | 98.8 | 100 |
| Mebendazole | 78.0 | 50.0 | 5.4 | 11.1 |
| Metformin HCl | 100 | 50.0 | 100 | 75.0 |
| Metitepine mesylate | 69.8 | 88.9 | 100 | 100 |
| Morantel citrate | 60.3 | 47.2 | 100 | 94.4 |
| Moxidectin | 100 | 100 | 9.6 | 27.8 |
| Natamycin | 95.2 | 52.8 | 100 | 61.1 |
| Phenothiazine | 98.3 | 83.3 | 0 | 23.8 |
| Phenylmercuric acetate | 100 | 100 | 100 | 100 |
| Pramoxine HCl | 100 | 22.2 | 75.1 | 16.7 |
| Pyrantel pamoate | 91.2 | 58.3 | 26.8 | 90.0 |
| Selamectin | 78.7 | 27.8 | 1.3 | ND |
| Tetramizole HCl | 100 | 86.1 | 100 | 100 |
| Thiabendazole | 93.2 | 0 | 94.9 | 33.3 |
| Thonzonium bromide | 97.7 | 61.1 | 100 | 100 |
| Trichlorfon | 100 | 100 | 100 | 100 |
| Triclosan | 97.6 | 52.8 | 100 | 100 |
| Triflupromazine HCl | 83.4 | 43.3 | 100 | 100 |
| Trimeprazine tartrate | 63.5 | 33.3 | 90.0 | 88.9 |
| Trimipramine maleate | 62.1 | 6.7 | 98.9 | 75.0 |
| Zoxazolamine | 98.4 | 27.8 | 6.9 | 4.8 |

**ND: not done**
